# *Andrias davidianus* Liver-Derived Peptides Ameliorate MASH Accompanied by Attenuation of PPARγ Signaling and Selective Modulation of Gut Microbiota

**DOI:** 10.3390/metabo16080532

**Published:** 2026-07-28

**Authors:** Xing Shen, Ya-Na Qu, Yi-Xiao Huang, Chen-Yu Liang, Si-Jia Liu, Na Liu, Zi-Jie He, Shuai-Kun Su, Qing-Zhu Sun, Tai An, Hua Han

**Affiliations:** 1 Department of Biomedicine, Institute of Future Agriculture, Northwest A&F University, Yangling 712100, China; 2 College of Animal Science and Technology, Northwest A&F University, Yangling 712100, China; 3 State Key Laboratory of Cancer Biology, Department of Biochemistry and Molecular Biology, Fourth Military Medical University, Xi’an 710032, China; 4 Tangdu Innovative Institute of Science and Technology, Tangdu Hospital, Fourth Military Medical University, Xi’an 710038, China

**Keywords:** *Andrias davidianus* liver-derived peptides, MASH, the PPARγ signaling pathway, lipid metabolism

## Abstract

Background: Metabolic dysfunction-associated steatohepatitis (MASH), the progressive stage of metabolic dysfunction-associated steatotic liver disease (MASLD), is characterized by hepatic steatosis, inflammation, and fibrosis, yet no specific therapy has been established. This study evaluated the therapeutic potential of Andrias davidianus liver-derived peptides (ALPs) in a mouse model of MASH. Methods: ALPs were prepared by enzymatic hydrolysis of fresh Andrias davidianus liver. A MASH model was induced in mice using a methionine- and choline-deficient diet (MRCD). ALP was administered via oral gavage, and its effects were assessed through histological staining (H&E, Oil Red O, and Sirius Red), immunofluorescence (Ki67), apoptosis detection (TUNEL), serum biochemistry, and RNA-sequencing of liver tissues. Gut microbiota composition was also analyzed. Results: ALP treatment significantly alleviated hepatic histopathological features, including steatosis, inflammation, and fibrosis. It reduced aberrant proliferation and apoptosis of hepatocyte-like cells, and markedly improved serum biochemical markers of liver function. RNA-seq analysis revealed that ALP modulated the expression of lipid metabolism-related genes, an effect associated with suppression of the PPARγ signaling pathway. Furthermore, ALP selectively modulated MRCD-induced gut microbiota dysbiosis, particularly by reducing the Firmicutes-to-Bacteroidota (F/B) ratio and enriching *Akkermansia*. No overt toxicity was observed in other organs. Conclusions: Our findings demonstrate that ALP exerts protective effects against MASH by improving lipid metabolism, partially through suppression of the PPARγ signaling pathway, and by selectively modulating specific gut microbial taxa. ALP represents a promising natural therapeutic candidate for MASH.

## 1. Introduction

Metabolic dysfunction-associated steatotic liver disease (MASLD), previously known as non-alcoholic fatty liver disease (NAFLD), is characterized by the presence of hepatic steatosis (liver fat accumulation) often accompanied by obesity, insulin resistance, hypertension, dyslipidemia, among others [1]. With the changes in lifestyle and environment globally, MASLD incidences are experiencing a surge in prevalence: current data show that 38% of adults and 7–14% of children and adolescents suffer from this disease [2]. MASLD encompasses a series of metabolic dysfunction-associated diseases ranging from simple hepatic steatosis through cirrhosis, among which metabolic dysfunction-associated steatohepatitis (MASH) represents an actively progressive form of MASLD and is characterized by hepatic inflammation and fibrosis, in addition to steatosis [3]. The onset and progression of MASH have been a research focus for decades, and it is now clear that multiple risk factors and mechanisms, such as lipid toxicity, dysregulated reactive oxygen species (ROS) signaling, mitochondrial dysfunction, impaired protein homeostasis, as well as gut microbiota dysbiosis, are involved [4]. However, the clinical treatment of MASH has been limited to lifestyle interventions including dietary modifications, weight loss, physical activity [5], and to a few pharmacotherapies targeting MASH risk factors, such as vitamin E and pioglitazone, which is a peroxisome proliferator-activated receptor gamma (PPARγ) agonist. It is of note that these medications are associated with considerable safety concerns including weight gain, fluid retention, and potential carcinogenic risks [6].

In recent years, bioactive peptides, which are peptide mixtures prepared from plants or animals after proteolytic hydrolysis, have drawn considerable attention for their efficacy as functional ingredients in the treatment of chronic diseases [7]. Although the exact structure or consensus amino acid sequence of most biological peptides is still under investigation, their anti-inflammatory, antibacterial, antihypertensive, antioxidant roles as well as metabolic regulatory and immunomodulatory activities have been repeatedly reported [7,8,9,10]. For instance, bioactive peptides derived from seaweeds exhibit antihypertensive, antioxidant, and antidiabetic properties [10], while abalone peptides have been shown to enhance stress resistance and display anti-aging ability in *Caenorhabditis elegans* via the SKN-1-mediated metabolic reprogramming [11]. Moreover, protein hydrolysates from sardines and octopuses exert anti-hyperlipidemic effects [12,13], and protein hydrolysates from cuttlefish and tuna possess antihypertensive functions [14,15]. Importantly, compared with traditional therapeutic drugs, these peptides or protein hydrolysates have shown no obvious side effects, along with the advantages of wide availability and economic sustainability. They thus emerge as high-quality alternative approaches for preventing early-stage diseases and hold great application potential in the health field [9].

*Andrias davidianus* is the largest extant amphibian in the world and an important farmed animal in China. Evolutionarily, *Andrias davidianus* represents a transitional species from aquatic to terrestrial life. Various bioactive products have been prepared from *Andrias davidianus* for medical purposes. For instance, a composite hydrogel derived from *Andrias davidianus* has shown enhanced antibacterial and bone repair properties in test treatment of osteomyelitis [16]. More recently, *Andrias davidianus* peptide hydrogel has also shown beneficial effects on colitis by promoting efferocytosis [17], and biocompatible Pickering emulsions from *Andrias davidianus* byproducts for promoting burn wound healing [18]. In the current study, we show that bioactive peptides derived from *Andrias davidianus* liver hydrolysates (*Andrias davidianus* liver peptides, ALP) effectively ameliorate MASH in mice, which could be attributed to suppression of hepatic PPARγ signaling and remodeled gut microbiota.

## 2. Materials and Methods

**Mouse treatment and animal welfare.** Six-week-old male C57BL/6J mice were purchased from GemPharmatech Co., Ltd. (Nanjing, China). Mice were randomly assigned to three experimental groups (*n* = 6 per group): (1) normal control + saline (CON), (2) MRCD + saline (MRCD), and (3) MRCD + ALP (ALP). Mice were housed at a specific pathogen-free facility with constant temperature of 22 °C, a 12 h light/dark cycle, and free access to food and water. To induce MASH, mice were fed a methionine-restricted choline-deficient (MRCD) diet (XTMRCD60, Xietong Pharmaceutical Bioengineering Co., Ltd., Nanjing, China) for 4 weeks, with conventional chow-fed mice serving as controls. This commercial diet is formulated to match Research Diets formulation A06071302, containing 39.67% fat, 23.48% protein, and 26.77% carbohydrate by weight, which provides 60% kcal from fat (61.91% from fat, 17.79% from protein, and 20.29% from carbohydrate), with 0.1% methionine and no choline. During the 4-week ALP treatment phase, all mice continued to have ad libitum access to the same MRCD to maintain consistent pathological stimuli throughout the intervention.

The ALP was provided by Shaanxi Hua Ni Biotechnology Co., Ltd. (Xi’an, China), which was prepared by proteolytic hydrolysis of fresh liver from farmed adult *Andrias davidianus*, followed by isolating peptides of 12–30 amino acid residues as confirmed by high performance liquid chromatography (HPLC), and commercialized as a functional gradient. The peptide concentration of ALP preparations was determined by absorbance at A280. The detailed peptide sequences of this product fall within the patent-protected proprietary information of the company. For the batches used in this study, the company performed quality control using both HPLC fingerprinting and A280 absorbance to ensure consistency of the experimental material. To treat with ALP, mice were given daily ALP (8 mg/kg/day) by gavage for 4 weeks, whereas the control group received normal saline by gavage. The use of *Andrias davidianus* products in research complied with the Wildlife Protection Law of the People’s Republic of China. The protocol for animal experiments was reviewed and approved by the Institutional Animal Care and Use Committee (IACUC) of Northwest A&F University. All animal experiments were performed in strict accordance with the issued guidelines and regulations, and fully complying with the relevant ethical requirements.

**Histology.** Liver samples were fixed with 4% paraformaldehyde (PFA), embedded in paraffin, and sectioned at 4 μm thickness for hematoxylin and eosin (H&E) staining and Sirius Red staining following standard protocols. Otherwise, samples were embedded in optimal cutting temperature compound (OCT) and cryo-sectioned into 5 μm thick slices, followed by Oil Red O staining according to the standard protocol. Images were captured under an optical microscope (Leica Biosystems, Wetzlar, Germany). To evaluate proliferation and apoptosis in hepatocyte-like cells, sections were stained with a Ki67 antibody kit and TUNEL assay kit (both from Beyotime Biotechnology, Shanghai, China), respectively, and were observed using a confocal microscope (Leica). Images were quantitatively analyzed using the ImageJ software. All histological scorings—including NAS scoring and quantification of Oil Red O/Sirius Red-positive areas—were performed by an investigator blinded to group allocation. Tissue samples were coded prior to analysis, and group identities were revealed only after all measurements were completed.

**Transmission electron microscopy (TEM).** Liver tissues were dissected from mice and then immediately fixed in 2.5% glutaraldehyde at 4 °C. After washing, tissues were post-fixed in 2% osmium tetroxide, dehydrated in ethanol/propylene oxide and embedded in epoxy resin. Then, liver tissues were cut at 70 nm thickness using an ultramicrotome (Leica UC7), and then stained with uranyl acetate and lead solution. The sections were air-dried, and images were captured under a transmission electron microscope (HT7700, Hitachi, Tokyo, Japan), and analyzed using Image-Pro Plus 6.0 (Media Cybernetics, Rockville, MD, USA).

**Blood biochemistry.** Serum was collected by centrifugation at 3000 rpm for 15 min followed by 5000 rpm for 5 min at 4 °C. The alanine transaminase (ALT), aspartate transaminase (AST), total cholesterol (TC), triglycerides (TG), high-density lipoprotein (HDL) were measured using a biochemistry analyzer (Model 7200-202, Hitachi Ltd., Tokyo, Japan) following the manufacturer’s instructions.

**RNA isolation, reverse transcription and quantitative polymerase chain reaction (RT-qPCR).** Total RNA was extracted from liver samples using the Trizol reagent. The RNA concentration was determined by measuring the optical density at 260 nm. Complementary DNA (cDNA) was synthesized using a kit (Vazyme Biotech Co., Ltd., Nanjing, China). Quantitative real-time polymerase chain reaction (qPCR) was performed with the SYBR Premix Ex Taq (Takara Dalian, Dalian, China) and a QuantStudio 5 realtime PCR instrument (Life Technologies, Waltham, MA, USA) according to the manufacturers’ instructions. The relative expression levels of target genes were normalized to glyceraldehyde-3-phosphate dehydrogenase (GAPDH) and analyzed using the 2^−ΔΔCt^ method. PCR primers are shown in Table 1.

**Protein extraction and Western blotting.** Total liver proteins were extracted by homogenizing in the radio-immunoprecipitation assay (RIPA) lysis buffer (Beyotime Biotechnology, Shanghai, China), and supernatants were collected after centrifugation at 10,000× *g* for 15 min at 4 °C. The protein concentration was determined using the BCA Protein Assay Kit (Beyotime), and protein samples were then separated by sodium dodecyl sulfate-polyacrylamide gel electrophoresis (SDS-PAGE) and transferred onto polyvinylidene fluoride (PVDF) membranes by electroblotting. Membranes were then incubated with primary antibodies, followed by the corresponding secondary antibodies. The primary antibodies included PPARγ (Cat#AF6284, RRID:AB_2835135; Affinity Biosciences, Cincinnati, OH, USA) and CD36 (Cat# HA724000, RRID: AB_3740144; Huabio, Hangzhou, China). The secondary antibody was HRP-conjugated goat anti-rabbit IgG (Cat# SA00001-2, Sangon Biotech, Shanghai, China). Bands were developed using a chemoluminescence system (Tanon, Shanghai, China). The grayscale density of the protein bands was normalized to β-actin as an internal reference. Quantification was performed with the ImageJ 2× software (Rawak Software, Stuttgart, Germany).

**RNA sequencing.** Transcriptome analysis of mouse liver tissues was performed by Biomarker Technologies Co., Ltd. (Beijing, China). Following nucleic acid extraction using Trizol reagent, the concentration of the extracted nucleic acids was quantified with a NanoDrop 2000 spectrophotometer (Thermo Fisher Scientific, Waltham, MA, USA), and nucleic acid integrity was assessed using an Agilent 2100 Bioanalyzer combined with the LabChip GX system (PerkinElmer, Hopkinton, MA, USA). After the samples passed the quality inspection, library construction was carried out. Upon completion of library preparation, an initial quantification was conducted using a Qubit 3.0 Fluorometer (Invitrogen, Thermo Fisher Scientific, Waltham, MA, USA), with the required concentration reaching ≥1 ng/μL. Subsequently, the insert size of the libraries was examined using the Qsep400 High-throughput Analysis System (Bioptic Inc., New Taipei City, China). Once the insert size met the expected criteria, quantitative polymerase chain reaction (q-PCR) was employed for accurate determination of the effective library concentration, which was required to be >2 nM to ensure library quality. Ultimately, the qualified libraries were subjected to high-throughput sequencing on an Illumina NovaSeq 6000 platform. Bioinformatics analysis utilized OmicShare tools (www.omicshare.com/tools, accessed on 18 August 2025), TB Tools, and GSEA2.2.4. Data are available in the Genome Sequence Archive (GSA; accession PRJNA1449774).

**Targeted sequencing of bacterial 16S rDNA.** For gut microbiota analysis, mouse fecal samples were randomly selected and processed by LC-Bio Technologies Co., Ltd. (Hangzhou, China). Briefly, total DNA was extracted from fecal samples using the Fecal Genomic DNA Extraction Kit (Cat. No. AU46111-96, BioTeke Corporation, Beijing, China) in accordance with the manufacturer’s instructions. Using the extracted total DNA as the template, PCR amplification was performed. After quality assessment of the qualified PCR amplicons, pooled library construction was conducted. Sequencing was then accomplished on the Illumina NovaSeq 6000 platform with paired-end 250 bp sequencing. Bioinformatics analysis utilized OmicShare tools (www.omicshare.com/tools (accessed on 20 July 2026)), TB Tools, and GSEA2.2.4. Data are available in the Genome Sequence Archive (GSA; accession PRJNA1449773).

**Statistical analysis.** Data were analyzed with FlowJo V.10 and GraphPad Prism 8.0. Quantitative data are expressed as mean ± SEM. All experiments were performed with six mice per group (*n* = 6 per group). Differences between two groups were evaluated using unpaired two-tailed *t*-tests, while multiple groups were compared by one-way ANOVA with Tukey’s post hoc test. *p* < 0.05 was considered statistically significant.

## 3. Results

### 3.1. ALP Ameliorates General MASH Phenotypes in MRCD-Induced Mice

**Figure 1:**
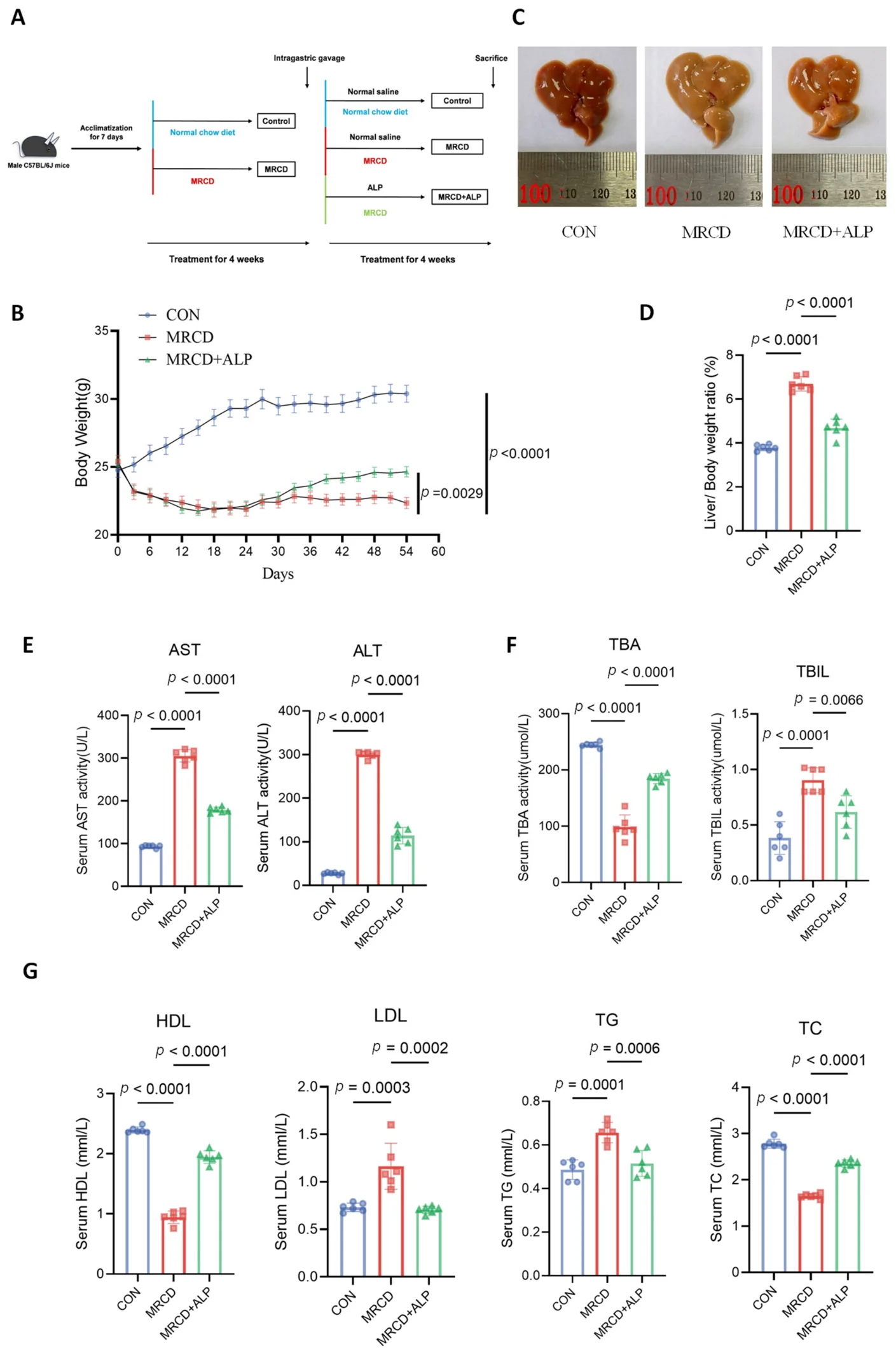
**Effects of ALP administration on MASH in a mouse model.** (**A**) Schematic illustration of the experimental design. (**B**) Body weight changes in mice across experimental groups. (**C**) Representative morphological images of livers from different groups of mice. (**D**) Liver index (liver weight/body weight × 100) of mice in different groups. (**E**–**G**) Serum levels of aspartate aminotransferase (AST) and alanine aminotransferase (ALT) (**E**), total bile acid (TBA) and total bilirubin (TBIL) (**F**), as well as serum lipid profiles, including high-density lipoprotein cholesterol (HDL-c), low-density lipoprotein cholesterol (LDL-c), triacylglycerol (TG), and total cholesterol (TC) (**G**), in mice of different experimental groups. Data are mean ± SD; one-way ANOVA with Tukey’s post hoc test. *n* = 6 mice per group.

To investigate the effects of ALP treatment on MASH, we first established the MASH animal model using the MRCD in C57BL/6J mice, with mice fed the standard chow diet as the control group (CON). After 4 weeks of the MRCD, some of the MASH mice were treated with ALP by gavage for 4 more weeks, while the CON and untreated MRCD groups received an equal volume of normal saline (Figure 1A). Body weight monitoring indicated that while the MRCD-fed mice exhibited a rapid decline in body weight as compared with the CON group, MASH mice treated with ALP showed significantly restored body weight as compared with the untreated MASH group (Figure 1B). Consistently, compared with the CON group, livers from the MRCD group displayed prominent pathological features, including marked yellowing, volume enlargement, and a greasy surface texture. In contrast, livers from the ALP-treated group exhibited a notably improved appearance and morphology (Figure 1C). Moreover, the liver weight index (liver/body weight in percentage) of the MRCD group was significantly elevated compared with the CON group, which was markedly reduced in the ALP-treated group as compared with the MRCD group (Figure 1D).

Serum biochemical analysis revealed that compared with the CON group, the MRCD group exhibited significantly elevated levels of serum aspartate aminotransferase (AST) and alanine aminotransferase (ALT). ALP treatment significantly reduced both of these liver injury markers (Figure 1E). Concurrently, ALP treatment reduced serum total bile acid (TBA) and total bilirubin (TBIL) levels in the MRCD-induced MASH group (Figure 1F). Furthermore, ALP treatment effectively improved MRCD-induced dyslipidemia, as shown by the restored serum levels of high-density lipoprotein cholesterol (HDL), total cholesterol (TC), low-density lipoprotein cholesterol (LDL), and triglycerides (TG) (Figure 1G). These results suggest that ALP treatment could mitigate MASH in mice.

### 3.2. ALP Treatment Ameliorates MRCD-Induced Hepatic Histopathological Injury and Lipid Deposition

**Figure 2:**
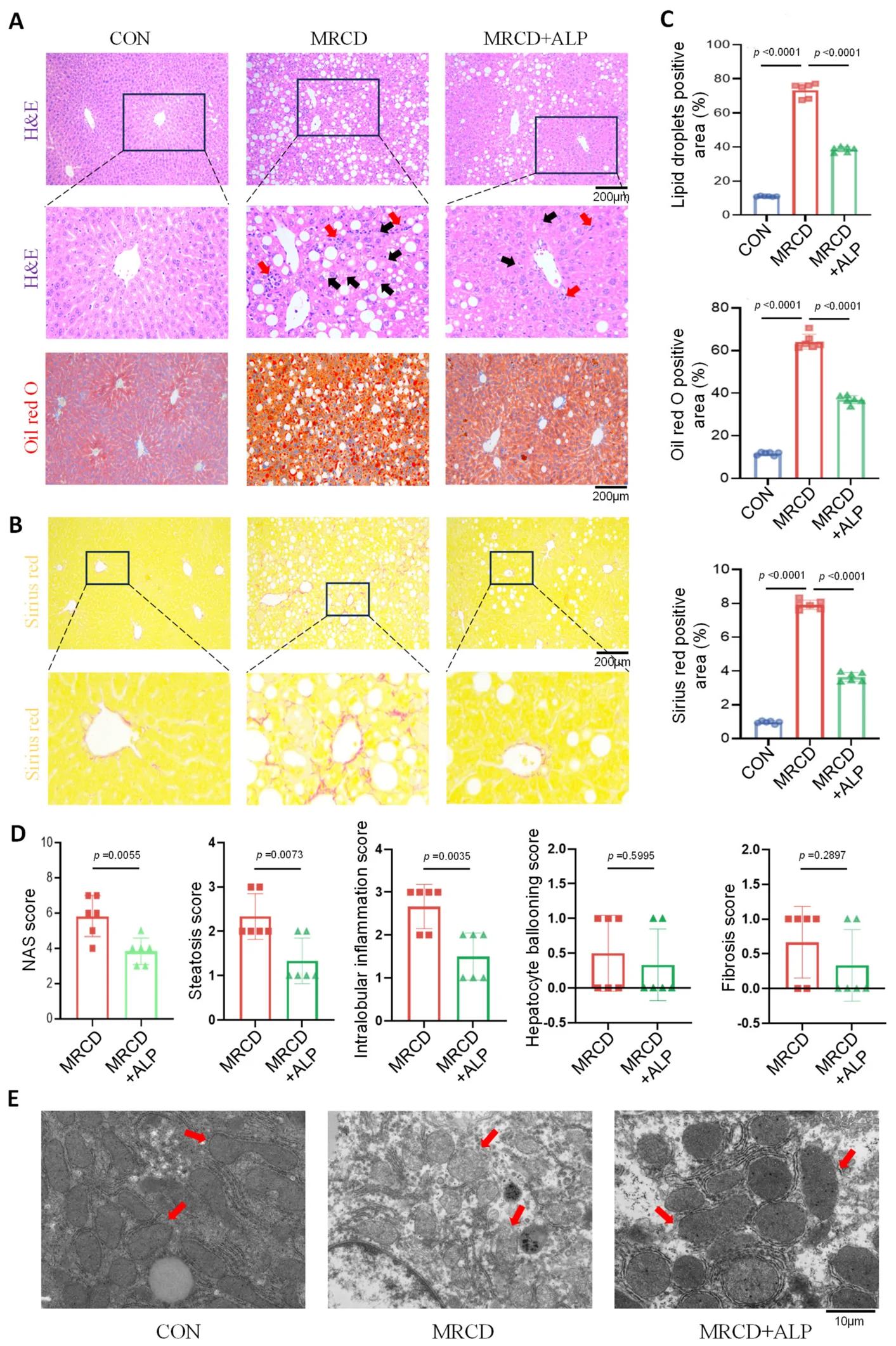
**ALP ameliorates hepatic steatosis and fibrosis in MRCD-induced MASH mice.** (**A**) Representative images of H&E staining (**upper panels**) and Oil Red O staining (**lower panels**) of liver sections from different groups. Scale bars: 200 μm (H&E) and 200 μm (Oil Red O). For H&E staining, the insets (dashed boxes in the corresponding panels) show higher-magnification views of selected areas. In the magnified insets, red arrows mark lobular inflammation and black arrows mark ballooned hepatocytes. (**B**) Representative photomicrographs of Sirius Red staining of liver tissues from each group (scale bar, 200 μm). (**C**) Quantitative analysis of Oil Red O-positive areas (representing lipid droplets) and Sirius Red-positive areas (representing collagen fibers) in liver tissues across all groups. (**D**) NAFLD/MASLD activity score (NAS, evaluated by steatosis, lobular inflammation, and hepatocellular ballooning) and fibrosis score for each group. (**E**) Representative transmission electron microscopy images of liver tissue from each group (scale bars = 10 μm. Arrows denote mitochondria). Data are mean ± SD; one-way ANOVA with Tukey’s post hoc test. *n* = 6 mice per group.

We then examined liver histopathology in MRCD-induced MASH mice treated with ALP. H&E staining revealed that the MRCD group displayed disorganized hepatic cords, extensive steatosis, and significant inflammatory cell infiltration. ALP treatment markedly alleviated these histopathological injuries (Figure 2A). Oil Red O staining indicated a significant increase in hepatic lipid deposition in the MRCD group, whereas ALP treatment significantly reduced the lipid droplet areas (Figure 2A). Sirius Red staining showed a pronounced collagen fiber deposition in the MRCD group, which was significantly reduced after ALP treatment (Figure 2B). Quantitative analysis demonstrated that the hepatic lipid deposition area reached 64% in the MRCD group but decreased to 37% in the ALP-treated group (Figure 2C). Evaluation of liver tissue changes with the NAFLD/MASLD activity score (NAS) showed that ALP treatment reduced steatosis scores (MRCD: 2.33 ± 0.52 vs. ALP: 1.33 ± 0.52), lobular inflammation scores (MRCD: 2.67 ± 0.52 vs. ALP: 1.50 ± 0.55), hepatocyte ballooning scores (MRCD: 0.50 ± 0.55 vs. ALP: 0.33 ± 0.52), and fibrosis scores (MRCD: 0.67 ± 0.52 vs. ALP: 0.33 ± 0.52), resulting in a decreased total NAS in the ALP-treated group (Figure 2D). Furthermore, transmission electron microscopy (TEM) revealed slightly swollen mitochondria with partially fragmented and reduced cristae, and partial dissolution of the intra-matrix content in the MRCD group. ALP treatment improved mitochondrial structure, resulting in less swelling and more organized mitochondrial cristae (Figure 2E). Collectively, these results support the protective role of ALP against MASH by reducing hepatocyte steatosis, lipid deposition, and mitochondrial damage.

### 3.3. ALP Reverses Aberrant Hepatocyte-like Cell Proliferation and Excessive Apoptosis in MRCD-Induced MASH Mice

**Figure 3:**
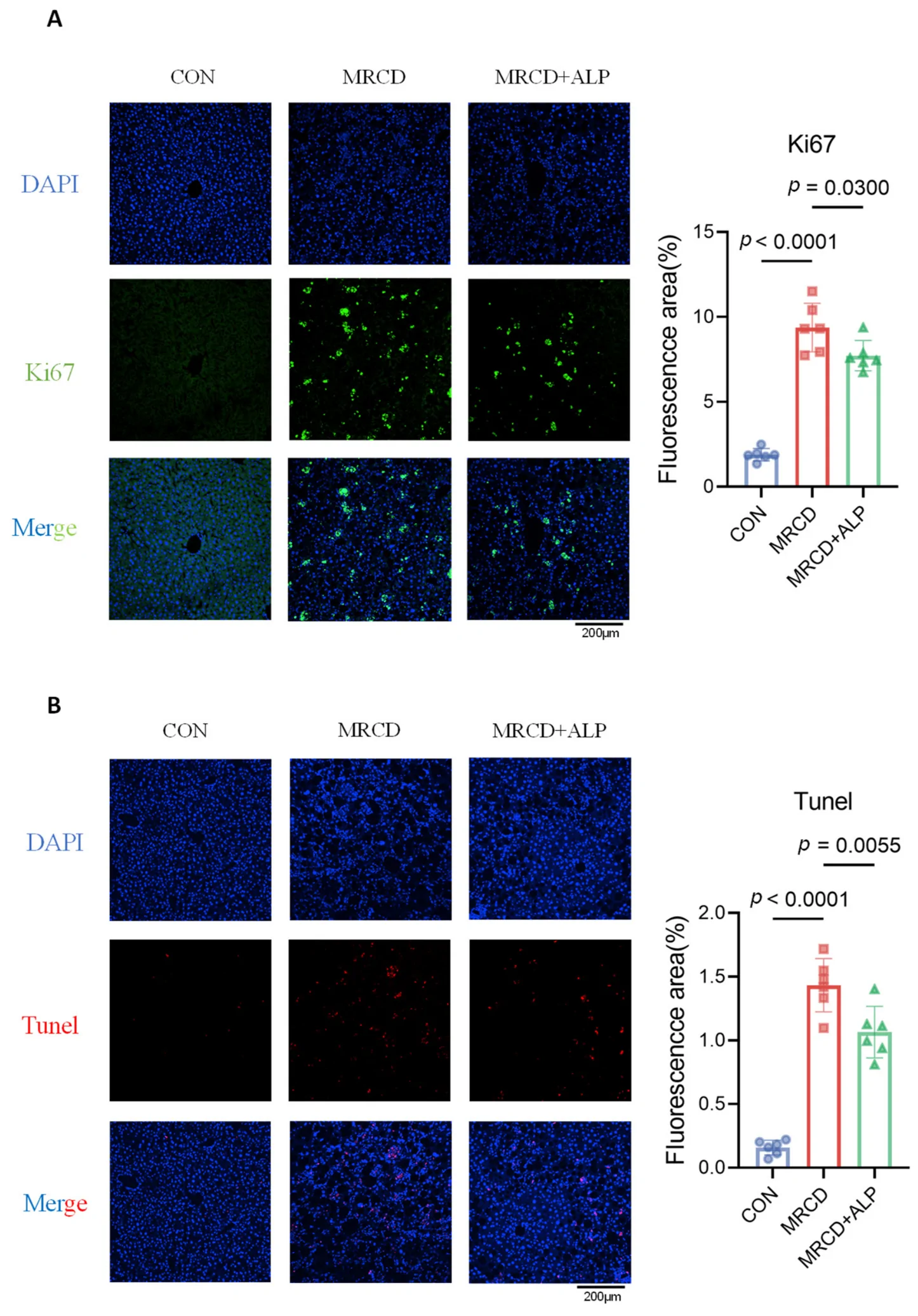
**Effects of ALP on hepatocyte proliferation and apoptosis in MRCD-induced MASH mice.** (**A**) Representative immunofluorescence images (**left**) and quantitative analysis (**right**) of the proliferation marker Ki67 (green) in hepatocyte-like cells in liver sections from the indicated groups. Nuclei were counterstained with DAPI (blue). (**B**) Representative images (**left**) and quantitative analysis (**right**) of apoptosis detected by the TUNEL assay (green) in hepatocyte-like cells in liver sections from different groups. Nuclei were counterstained with DAPI (blue). Data are mean ± SD; one-way ANOVA with Tukey’s post hoc test. *n* = 6 mice per group.

MASH triggers hepatocyte proliferation and excessive apoptosis. We examined the proliferation and apoptosis of hepatocyte-like cells by Ki67 immunofluorescence staining and TUNEL staining, respectively. Ki67- and TUNEL-positive cells were identified as hepatocyte-like cells based on their characteristic morphology (large polygonal shape, centrally located round nuclei, and abundant cytoplasm). The results showed that compared to the control group (CON, Ki67 fluorescence intensity: 1.89 ± 0.37), the MRCD group exhibited significantly enhanced proliferation of hepatocyte-like cells (9.37 ± 1.43, *p* < 0.0001), while ALP treatment significantly reduced this aberrant proliferation of hepatocyte-like cells (7.72 ± 0.89, *p* < 0.05) (Figure 3A,B). Similarly, TUNEL staining revealed that apoptosis of hepatocyte-like cells was markedly increased in the MRCD group (1.43 ± 0.21) compared with the CON group (0.16 ± 0.06), and ALP treatment significantly attenuated this excessive apoptosis of hepatocyte-like cells (1.07 ± 0.20) (Figure 3A,B). These findings suggest that ALP could regulate the proliferation and apoptosis of hepatocyte-like cells directly or indirectly in MRCD-induced MASH mice.

### 3.4. ALP Ameliorates MRCD-Induced MASH Accompanied by Attenuated PPARγ Signaling

**Figure 4:**
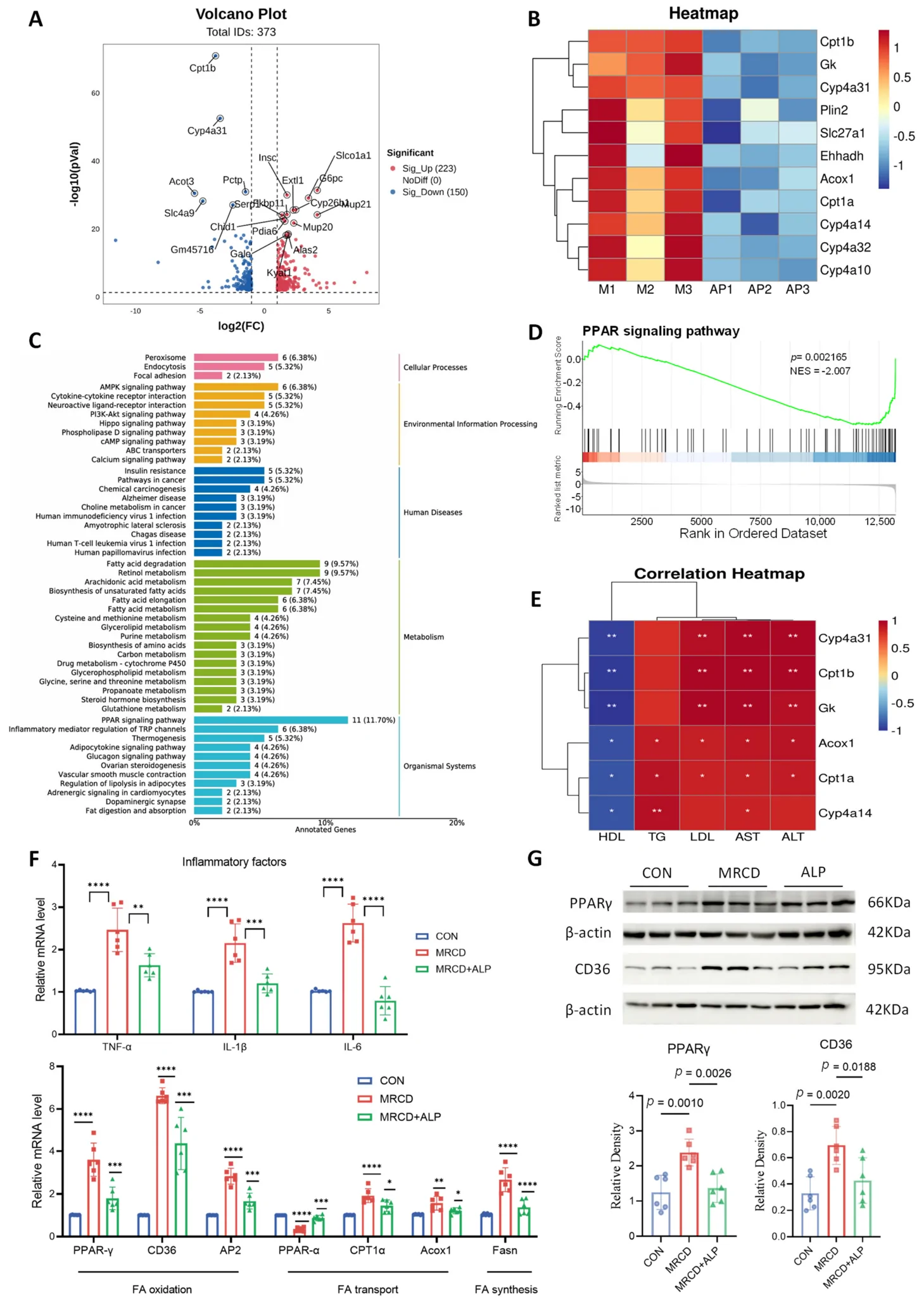
**Transcriptomic analysis and validation of PPARγ-related pathways in the livers of ALP-treated MASH mice.** (**A**) Volcano plot of differentially expressed genes (DEGs). A total of 373 DEGs were identified, including 223 upregulated and 150 downregulated genes. (**B**) Heatmap of key genes involved in the PPARγ signaling pathway. (**C**) KEGG enrichment analysis of the PPARγ signaling pathway. (**D**) Gene Set Enrichment Analysis (GSEA) of the PPARγ signaling pathway. (**E**) Correlation heatmap between key metabolic genes and serum biochemical parameters (HDL, TG, LDL, AST, ALT), where red indicates positive correlations and blue indicates negative correlations. (**F**) Relative mRNA expression levels of inflammatory factors (TNF-α, IL-1β, IL-6) and fatty acid metabolism-related genes (PPARγ, CD36, CPT1a, etc.) in liver tissues from different groups. (**G**) Western blotting analysis of PPARγ and CD36 protein expression and quantification, showing that MRCD treatment significantly increased their expression, and this increase was reversed by ALP treatment. Data are mean ± SD; one-way ANOVA with Tukey’s post hoc test. *n* = 6 mice per group. *: *p* ≤ 0.05; **: *p* ≤ 0.01; ***: *p* ≤ 0.001; ****: *p* < 0.0001.

To assess the potential mechanism mediating the effects of ALP on the MRCD-induced MASH in mice, we performed RNA-seq analysis using liver tissue samples from ALP-untreated and -treated MRCD mice. Principal component analysis (PCA) showed distinct clustering of the two groups. A comparison of gene expression identified 373 significantly differentially expressed genes (DEGs) between the ALP-untreated and ALP-treated groups. Among these, Cpt1b and Cyp4a31, which were targets of the PPAR signaling pathway, were the most significantly downregulated genes (Figure 4A,B). KEGG pathway enrichment analysis indicated that DEGs were primarily enriched in metabolic pathways including the PPAR signaling pathway and fatty acid metabolism. Notably, the PPAR signaling pathway showed a high degree of enrichment, suggesting it may be one of the key pathways regulated by the ALP treatment (Figure 4C). Gene Set Enrichment Analysis (GSEA) further revealed that the PPAR signaling pathway was significantly attenuated in the ALP-treated group as compared with the ALP-untreated MRCD group (NES = −2.0007, *p* < 0.001). Correlation analysis between core DEGs and serum markers showed that genes such as Acox1 and Cpt1a were significantly positively correlated with TG, AST, and ALT levels (Figure 4E).

To confirm the expression levels of DEGs in the livers of mice following various treatments, we performed RT-qPCR and Western blotting analysis. RT-qPCR showed that compared with the CON group, the mRNA expression levels of inflammatory cytokines TNF-α, IL-1β, and IL-6 in the liver tissue of the MRCD group were significantly upregulated; these were downregulated upon ALP treatment (Figure 4F). Concurrently, the mRNA expression of PPAR signaling pathway-related target genes, PPARγ and CD36, was abnormally elevated in the MRCD group, and the ALP treatment effectively downregulated their expression levels (Figure 4F). Western blotting analysis further confirmed that PPARγ and CD36 were upregulated in the liver of MRCD-induced MASH mice; these were downregulated by ALP treatment (Figure 4G). In summary, ALP treatment could ameliorate MRCD-induced MASH phenotypes, which is associated with the attenuation of the PPAR signaling pathway.

### 3.5. Effect of ALP Treatment on Gut Microbiota in the MASH Model

**Figure 5:**
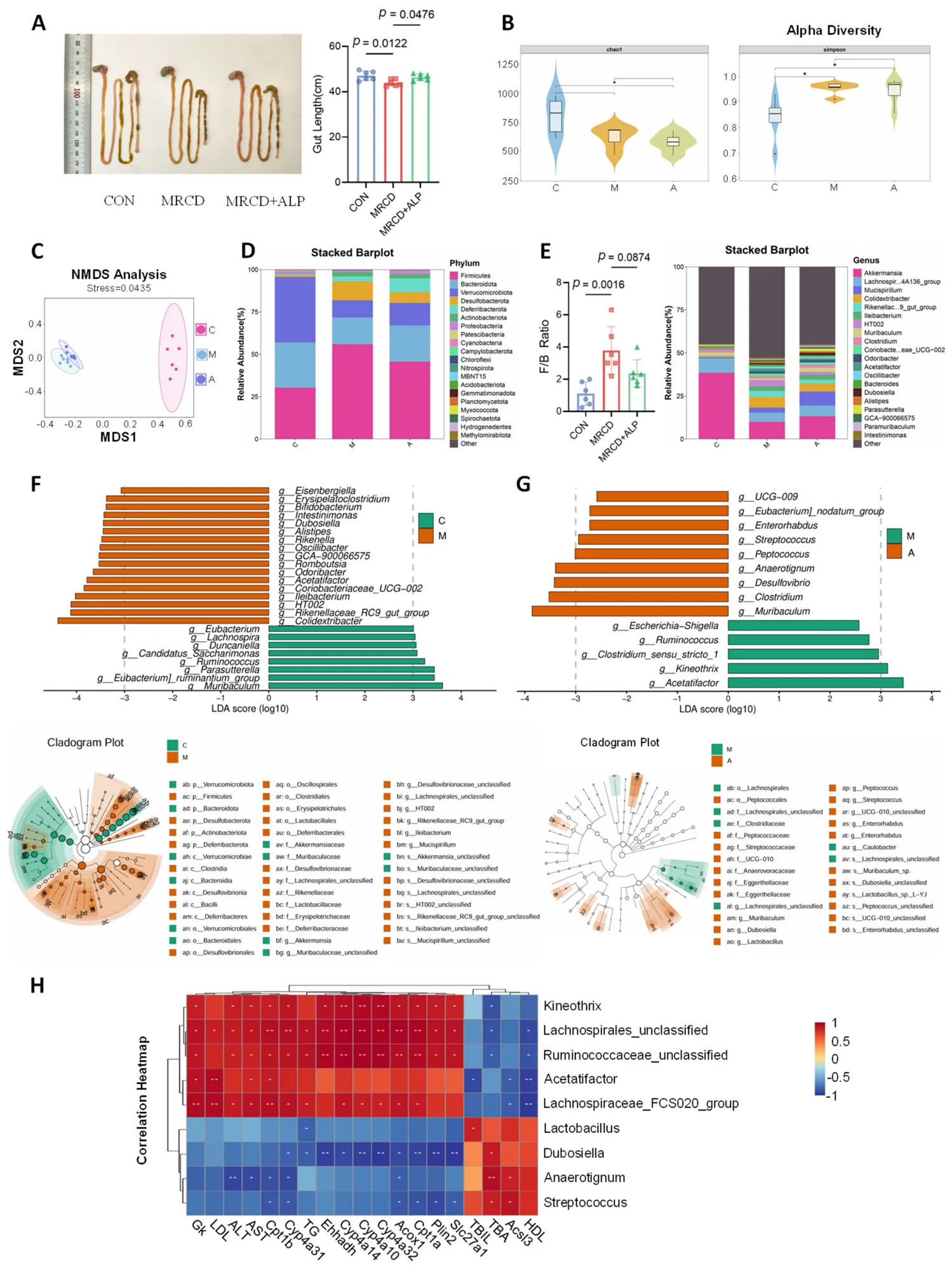
**Effects of ALP intervention on intestinal morphology and gut microbiota in MRCD-induced MASH mice.** (**A**) Photographs of intestinal macroscopic morphology and quantitative analysis of intestinal length in each group. (**B**) Alpha diversity analysis of gut microbiota based on Chao1 and Shannon indices. (**C**) NMDS analysis based on Bray–Curtis distance, showing beta diversity differences in gut microbiota among groups. (**D**) Stacked bar plots of gut microbiota relative abundance at the phylum level (**left**) and genus level (**right**). (**E**) Quantitative analysis of the *Firmicutes/Bacteroidetes* (F/B) ratio in gut microbiota of each group. (**F**,**G**) LEfSe analysis of differential microbiota between the CON and MRCD groups and between the MRCD and MRCD + ALP groups (LDA score > 4). (**H**) Spearman correlation heatmap between gut microbiota and serum metabolic parameters. Colors indicate the magnitude of correlation coefficients, and *: *p* ≤ 0.05; **: *p* ≤ 0.01. Data are mean ± SD; one-way ANOVA with Tukey’s post hoc test. *n* = 6 mice per group.

The gut–liver axis represents the mutual interaction between the gut and liver. The gut microbiota and its metabolites profoundly influence physiological and pathological processes in the liver, and vice versa [19,20]. The total length of the gut was shortened in the MRCD-induced MASH mice, which was reversed by ALP treatment (Figure 5A). We then examined alterations in gut microbiota in ALP-treated MASH mice using 16S rDNA sequencing. The results showed that, compared with the CON group, the MRCD group exhibited decreased Chao1 index and an increased Simpson index, which were barely influenced by the ALP treatment (Figure 5B). Moreover, non-metric multidimensional scaling (NMDS) analysis showed clear separation between the CON and MRCD groups, and the ALP treatment showed negligible influence, suggesting that ALP treatment might not modulate the structural composition of the gut microbiota in MASH mice (Figure 5C).

A comparison of the top 20 species annotation results showed that the dominant phyla in the CON group were *Firmicutes*, *Bacteroidota*, and *Verrucomicrobiota* (Figure 5D), but the MRCD group exhibited an increased *Firmicutes*-to-*Bacteroidota* (F/B) ratio resulting from elevated *Firmicutes* abundance and decreased *Bacteroidota* and *Verrucomicrobiota* abundances (Figure 5E). The ALP treatment reduced the F/B ratio (Figure 5E). At the genus level (selecting the top 20 genera), compared with the CON group, the MRCD group exhibited a decreased relative abundance of *Akkermansiaceae*, which was reversed by the ALP treatment. Moreover, in the MRCD group, genera such as *Colidextribacter*, *Rikenellaceae*_RC9_gut_group, and *Ileibacterium* had LDA discriminant scores > 3 as evaluated by the Linear Discriminant Analysis Effect Size (LEfSe) (with an LDA score ≥ 3 set as the threshold for statistical significance), identifying them as core differentially enriched species in this group (Figure 5F,G). The cladogram further revealed that the differential species in the MRCD group were primarily concentrated within multiple clades under the phyla *Actinobacteria* and *Firmicutes*, indicating that MRCD treatment induced enrichment of specific microbial taxa at the phylogenetic level. In contrast, genera such as *Muribaculum*, *Clostridium*, and *Anaerotignum* in the ALP-treated group had LDA discriminant scores ≥ 3, serving as specifically enriched species for this group (Figure 5F,G). The cladogram showed that the differential species in the ALP-treated group were mainly distributed within specific taxonomic units of the phylum *Firmicutes* (Figure 5F,G), suggesting that ALP treatment exerted selective modulation on specific phylogenetic taxa of the gut microbiota in MASH mice, rather than inducing a global community-level shift. Correlation heatmap analysis between gut microbiota (genus level) and hepatic lipid metabolism as well as serum biomarkers showed that one class of microbes (*Kineothrix*, *Lachnospirales*, *Ruminococcaceae*, *Acetatifactor*, Group FCS020) showed significant positive correlations with liver injury (ALT, AST), lipid accumulation (TG, LDL), and PPAR signaling pathway-related genes (Cyp4a31, Cpt1b), and negative correlations with improved lipid metabolism and bile acids (HDL, Acsl3, TBA), while the other class (*Lactobacillus*, *Dubosiella*, *Anaerotignum*, *Streptococcus*) exhibited opposite correlations (Figure 5H). These results indicate that MRCD treatment can induce the enrichment of specific microbial taxa, while the ALP treatment can reverse this microbial dysbiosis and promote the enrichment of potentially beneficial genera such as *Akkermansiaceae*.

## 4. Discussion

The liver serves as the central metabolic organ of the body, and accepts blood flow from both systemic and portal circulation [21,22]. These high metabolic loads make the liver vulnerable to abnormal metabolic inputs from both the gut and other organs such as adipose tissue, leading to MASLD [23]. Impaired hepatic metabolism arises from increased uptake of free fatty acids (FFA), defective lipogenesis and FFA oxidation, as well as reduced lipid export [24]. Excessive lipid accumulation within hepatocytes leads to overproduction of mitochondrial reactive oxygen species (ROS) [25]. Oxidative stress can trigger mitochondrial dysfunction and hepatocyte cytotoxicity, ultimately resulting in cell death. Hepatocyte death and liver injury induce the recruitment of immune cells and activation of pro-inflammatory pathways, which further aggravate liver damage and promote excessive release of pro-inflammatory cytokines (TNF-α, TGF-β, IL-6, IL-1β), making MASLD progress into MASH [26,27]. The complex etiology and pathology of MASLD/MASH have hampered the establishment of a specific therapy.

In this study, we evaluated the role of ALP derived from Andrias davidianus liver hydrolysis in the MASH model using the MRCD-induced mouse model. Our results have demonstrated that ALP administration significantly alleviated MASH progression as shown by hepatic histology. Serum biochemistry indicated that liver function was improved markedly. No pathological alterations were detected in other organs at the tissue level in ALP-treated mice. However, it should be noted that the MRCD-induced MASH model, while useful for rapid proof of concept screening, does not fully recapitulate the obese, insulin resistant phenotype typical of most human MASH patients, as it induces weight loss and relies on nutritional deficiencies rather than caloric excess [28]. Therefore, our findings require cautious interpretation when extrapolating to human MASH. These findings strongly suggest that ALP represents a potential therapeutic ingredient for MASH.

Marine bioactive peptides have been shown to mitigate liver diseases through various pathways. In most cases, the effective component of these peptide preparations is unclear. For instance, anglerfish peptide modulates hepatic antioxidant capacity via the AMPK and Nrf2 pathways, thereby alleviating NAFLD [29]. In contrast, some consensus peptides have been identified to act in specific pathways to exert their therapeutic effects. The Pacific oyster-derived peptide Leu-Gln-Pro-Pro-Arg activates alcohol dehydrogenase and improves alcoholic liver disease by promoting alcohol metabolism and inhibiting mitochondrial apoptosis [30]. Our RNA-seq of liver tissues showed that ALP treatment modulated lipid metabolism-related genes, which was associated with attenuation of the PPARγ signaling pathway. PPARs are fatty acid-activated transcription factors belonging to the nuclear hormone receptor superfamily and play crucial roles in glucose and lipid metabolism [31]. PPARγ is a core regulatory molecule involved in MASLD by orchestrating key pathological processes including dysregulated lipogenesis, the development of insulin resistance, activation of inflammatory pathways, disruption of oxidative stress balance, induction of endoplasmic reticulum stress, and the advancement of hepatic fibrosis [32]. It is therefore likely that ALP treatment ameliorates MASH by attenuating the PPAR signaling pathway, but further studies are required to demonstrate a causality of this pathway in ALP treatment.

The apparent paradox that ALP attenuates PPARγ signaling, whereas the PPARγ agonist pioglitazone is used clinically for MASH, can be reconciled by recognizing the tissue specific and context-dependent roles of PPARγ. In adipose tissue, PPARγ activation improves insulin sensitivity and enhances adiponectin production, conferring systemic metabolic benefits—effects that underpin the therapeutic benefit of pioglitazone [33]. Conversely, hepatic PPARγ overexpression has been causally linked to increased fatty acid uptake via CD36, enhanced de novo lipogenesis, and aggravated inflammation, collectively driving steatosis and disease progression [34,35]. Indeed, liver-specific PPARγ deletion has been shown to protect against diet-induced hepatic steatosis and improve insulin resistance, supporting the concept that hepatic PPARγ is predominantly pathogenic under lipotoxic conditions [34,36]. Thus, we speculate that the therapeutic action of ALP involves liver-selective suppression of hepatic PPARγ-driven lipid uptake and inflammatory programs, while potentially sparing adipose PPARγ activity. Nevertheless, we acknowledge that our current data are correlative rather than causal. Future studies employing co-treatment with a PPARγ agonist or liver-specific PPARγ knockdown models will be essential to validate the causal role of this pathway in ALP anti-MASH effects and to determine the tissue specificity of ALP action.

The mutual communication between the gut and liver, or the gut–liver axis, plays pivotal roles in the pathogenesis of MASLD [37,38]. Bioactive peptides from various origins have been shown to benefit human health by modulating gut microbiota [39]. In the current study, our results revealed that the MRCD-induced MASH mice showed alterations in gut microbiota abundance at the phylum level: the relative abundance of *Firmicutes* increased, while that of *Bacteroidota* and *Verrucomicrobiota* decreased, leading to an elevated F/B ratio. The treatment with ALP altered the relative gut microbiota abundance at the phylum level with increased *Bacteroidota* and *Verrucomicrobiota*, leading to a decreased F/B ratio. The F/B ratio is a core indicator reflecting the homeostasis of gut microbiota structure, and its changes are directly linked to host metabolism and intestinal barrier function. An imbalance in the F/B ratio is often accompanied by impaired intestinal barrier integrity and LPS translocation, thereby exacerbating inflammation and fibrosis in MASH [40,41]. The ALP treatment therefore tends to partially modulate the gut microbiota structure in MASH mice. At the genus level, MASH mice treated with ALP showed increased relative abundances of *Akkermansia* and *Lachnospiraceae*_NK4A136_group. Recent studies indicate that *Akkermansia* contributes positively to intestinal mucus secretion and mucosal barrier repair, and also ameliorates chronic inflammation and metabolic disorders in the host [42,43]. *Lachnospiraceae*_NK4A136_group, a member of the *Lachnospiraceae* family, helps restore gut microbiota homeostasis disrupted by antibiotics and exhibits restorative effects on the intestinal barrier in diet-induced obese mice [44,45]. Therefore, ALP treatment selectively modulates gut microbiota dysbiosis in the MRCD-induced MASH model, which could contribute to the therapeutic effects of ALP. However, while ALP treatment significantly altered specific taxa—particularly reducing the F/B ratio and enriching *Akkermansia*—alpha diversity indices were minimally affected and NMDS analysis showed only modest separation between the MRCD and MRCD + ALP groups, indicating that ALP did not induce a global restructuring of the gut microbial community. These observations are not contradictory; rather, they suggest that ALP exerts selective modulation of key microbial taxa rather than broad-spectrum alteration of the entire community. This pattern is commonly observed in dietary or natural product interventions, as individual bioactive compounds often influence particular bacterial populations that harbor specific metabolic or sensing pathways, without globally disrupting the established microbial ecosystem. Moreover, the correlations between ALP enriched taxa and improved biochemical parameters provide correlative evidence supporting a potential link between microbial changes and host metabolic improvements. Future fecal microbiota transplantation (FMT) experiments will be required to establish causality and determine whether ALP modulated microbiota alone is sufficient to confer hepatoprotection.

In summary, the current study provides a novel natural active ingredient for the treatment of MASLD. It also opens up a new avenue for the high-value utilization of processing by-products from *Andrias davidianus*, which are farmed at a considerable scale in various areas of China. ALP exerts anti-MASH effects likely through different mechanisms including lipid metabolism improvement and gut microbiota remodeling. However, further studies are required to precisely identify the active component(s) of ALP and validate its therapeutic effect using clinical samples. Several additional limitations should be acknowledged. The ALP dose of 8 mg/kg/day was selected based on preliminary pilot experiments (6 and 8 mg/kg/day) and consultation with the manufacturer (HuaNi Biotechnology Co., Ltd., Xi’an, China), but without a formal dose-finding study or comprehensive dose–response design; therefore, we cannot determine whether this represents the optimal or minimally effective dose. In addition, the absence of a positive control group (e.g., pioglitazone) limits direct comparison of ALP efficacy with current standard of care pharmacotherapies. Furthermore, ALP used in this study is a proprietary product, and its detailed peptide sequences cannot be disclosed; the lack of LC MS/MS peptidomic characterization limits the identification of active component(s). We also note that Ki67-positive cells remained significantly more numerous in the MRCD group than in the normal control group. This is consistent with the known pathophysiological response in MASH, where chronic hepatocyte injury triggers compensatory regenerative proliferation [46]. ALP treatment significantly reduced, but did not completely normalize, this aberrant proliferation, suggesting that ALP attenuates the injury driven regenerative response without fully restoring homeostatic balance within the experimental timeframe. Future studies addressing these limitations—including alternative MASH models, dose–response assessments, positive controls, peptidomic characterization, and FMT validation—are currently planned as our highest priority research directions.

## 5. Conclusions

This study establishes ALP as a natural bioactive agent with dual therapeutic actions against MASH—hepatic PPARγ pathway attenuation and selective gut microbiota modulation. Unlike PPARγ agonists such as pioglitazone, which exert systemic effects with safety concerns, ALP appears to act through liver-selective suppression of PPARγ-driven lipogenic and inflammatory programs, offering a potentially safer regulatory strategy. Meanwhile, the enrichment of Akkermansia and restoration of the F/B ratio suggest that ALP engages the gut–liver axis as a complementary mechanism. These findings not only support ALP as a promising candidate for MASH intervention but also highlight the value of repurposing farmed Andrias davidianus byproducts for biomedical applications. Moving forward, integrating peptidomic identification, causality validation via liver-specific knockout models and FMT experiments, and dose-optimization studies will be essential to translate these preclinical observations into clinical therapeutics.

## Figures and Tables

**Table 1:** Primers used in this study.

Primer	Sequence (5′→3′)
β-actin-F	GAGACCTTCAACACCCCAGC
β-actin-R	ATGTCACGCACGATTTCCC
m-GAPDH-F	CACCATCTTCCAGGAGCGAG
m-GAPDH-R	CCTTCTCCATGGTGGTGAAGAC
Cpt1-α-F	GACTCCGCTCGCTCATTCC
Cpt1-α-R	ACCAGTGATGATGCCATTCTTG
Ap2-F	AAGGTGAAGAGCATCATAACCCT
Ap2-R	TCACGCCTTTCATAACACATTCC
Acox1-F	CATGCACCATTGCCATTCGATA
Acox1-R	CGGGAAGAGTTTATACTGCGT
Cd36-F	ATGGGCTGTGATCGGAACTG
Cd36-R	TTTGCCACGTCATCTGGGTTT
PPARγ-F	GGAAGACCACTCGCATTCCTT
PPARγ-R	GTAATCAGCAACCATTGGGTCA
PPARα-F	AACATCGAGTGTCGAATATGTGG
PPARα-R	CCGAATAGTTCGCCGAAAGAA
Fasn-F	CTGCCTTCGGTTCAGTCTCTT
Fasn-R	AGGCCACTTGTGGGGAATAC
TNF-α-F	CCTCTCTCTAATCAGCCCTCTG
TNF-α-R	GAGGACCTGGGAGTAGATGAG
IL6-F	TACCACTTCACAAGTCGGAGGC
IL6-R	CTGCAAGTGCATCATCGTTGTTC
IL1B-F	TCCAGGATGAGGACATGAGCAC
IL1B-R	GAACGTCACACACCAGCAGGTTA

## Data Availability

The data presented in this study are openly available in the Genome Sequence Archive (GSA) at https://ngdc.cncb.ac.cn/gsa/, (accessed on 20 July 2026), reference number PRJNA1449774 and PRJNA1449773. [Genome Sequence Archive (GSA)] [https://ngdc.cncb.ac.cn/gsa/ (accessed on 20 July 2026)] [PRJNA1449774 and PRJNA1449773].
